# Three in a Bed: Can Partner Support Improve CPAP Adherence? A Systematic Review and Intervention Recommendations

**DOI:** 10.3390/jpm15050192

**Published:** 2025-05-08

**Authors:** Giada Rapelli, Carola Caloni, Francesca Cattaneo, Marco Redaelli, Roberto Cattivelli, Giulia Landi, Eliana Tossani, Silvana Grandi, Gianluca Castelnuovo, Giada Pietrabissa

**Affiliations:** 1 Department of Psychology, University of Bologna, 40127 Bologna, Italy; giada.rapelli@unibo.it (G.R.); roberto.cattivelli@unibo.it (R.C.); giulia.landi7@unibo.it (G.L.); eliana.tossani2@unibo.it (E.T.); silvana.grandi@unibo.it (S.G.); 2 Department of Psychology, Catholic University of Milan, 20123 Milan, Italy; caloni.carola@gmail.com (C.C.); francescacattaneo98@yahoo.it (F.C.); marcoredaelli19@yahoo.it (M.R.); gianluca.castelnuovo@auxologico.it (G.C.); 3 Clinical Psychology Research Laboratory, IRCCS Istituto Auxologico Italiano, 20145 Milan, Italy

**Keywords:** obstructive sleep apnea syndrome, continuous positive airway pressure, adherence, couple, partner support

## Abstract

**Background/Objectives**: Continuous positive airway pressure (CPAP) is the standard approach for treating obstructive sleep apnea syndrome (OSAS), but patient adherence is often low due to various influencing factors. Recently, researchers have increasingly begun to explore the influence of partner support on adherence to CPAP therapy. This systematic review seeks to consolidate current evidence regarding the impact of partner support on CPAP adherence in individuals with OSAS. **Methods**: A comprehensive literature search was carried out across PubMed, Scopus, Medline, PsycINFO, and Web of Science databases under PRISMA guidelines. Stringent inclusion criteria were used, and at least two independent reviewers screened all studies. The mixed methods appraisal tool (MMAT) was used to assess selected articles for quality. Data relevant to the review’s objectives were extracted and presented through narrative synthesis. The review protocol was preregistered (Prospero CRD420251016574). **Results**: Nine studies met the inclusion criteria. Findings highlighted the significant influence of adherence to CPAP. Partner support, relationship quality, and collaborative efforts emerged as facilitators of adherence, with partnered individuals exhibiting higher adherence to CPAP use. However, barriers such as anxiety, interruption in intimacy, and conflict in relationships were also identified. **Conclusions**: To the best of our knowledge, this is the first systematic review to synthesize evidence on the partner’s role in CPAP adherence and inform clinicians on the importance of providing personalized care based on biopsychosocial characteristics of patients; for example, assessing the partner support in the management of the illness. Furthermore, the findings emphasize the need for further research—particularly randomized controlled trials and dyadic designs—to deepen understanding of how partner dynamics influence effects of CPAP treatment.

## 1. Introduction

Obstructive sleep apnea syndrome (OSAS) is a condition marked by irregular breathing during sleep, involving repeated episodes in which the upper airway is partially or completely blocked. These obstructions result in intermittent drops in oxygen levels, disrupted sleep, and overall poorer sleep quality [1,2].

This chronic condition affects a significant proportion of the adult population worldwide and represents a substantial public health concern due to its high morbidity and underdiagnosis [3].

Common clinical manifestations include loud snoring, witnessed apneas, nocturnal awakenings, and excessive daytime sleepiness, all of which can severely impact daily functioning and overall well-being [4].

If left untreated, OSAS can lead to a wide range of negative health outcomes, including cognitive, attentional, and memory impairments [5,6], mood disturbances such as depression and anxiety, and a marked deterioration in quality of life [7]. Moreover, OSAS significantly increases the risk for serious cardiovascular and metabolic conditions, including hypertension, stroke, myocardial infarction, and type 2 diabetes, as well as elevated mortality rates [8]. Indeed, these effects are further compounded by the intricate epidemiological and pathophysiological links between OSAS and obesity. Recently, much research has concentrated on weight loss as a strategy for managing OSAS [9].

The first-line treatment for OSAS is continuous positive airway pressure (CPAP), a non-invasive therapy that maintains airway patency by delivering a constant stream of pressurized air through a nasal or facial mask. When used consistently, CPAP has been shown to effectively reduce apnea–hypopnea events, improve sleep architecture and oxygenation, and lead to significant improvements in blood pressure regulation [10,11], neurocognitive performance, psychological well-being, and daytime alertness [12,13,14].

Despite its proven efficacy, long-term adherence to CPAP remains a significant challenge and is widely recognized as one of the primary barriers to effective OSAS management. Non-adherence is particularly critical during the early phases of therapy—often within the first few weeks or months—when patients are still adjusting to the physical and psychological demands of using the device [15,16,17]. A variety of factors have been identified as potential predictors of poor adherence, including demographic characteristics (e.g., age, gender) [18], technical discomfort or difficulties with the device [19], severity of the disorder [20], and emotional or psychosocial challenges [7]. Another aspect that contributes to the complexity of adherence is the lack of a clear definition of adherence in the context of CPAP therapy and the different assessments that could be implemented to measure it (self-report vs. using provided software). In fact, adherence refers to consistent use of the CPAP machine. However, the specific amount of nightly use needed for therapeutic benefit remains uncertain. Recommendations suggest using CPAP for 6–8 h per night, yet adherence criteria vary widely. Some studies define adherence as averaging 4 h per night on 70% of nights [21]. Due to these differing definitions, reported CPAP adherence rates range significantly, from as low as 28% to over 83% [21,22,23].

Given these complexities, promoting sustained CPAP use requires a multifaceted understanding of both the barriers and the enablers influencing patient behavior. Recent research has begun to shed light on the importance of social and interpersonal factors in shaping CPAP adherence. For example, patients have highlighted the need for structured education about OSAS and the benefits of CPAP, as well as ongoing support from healthcare professionals and peers with lived experience [24]. Many report feeling isolated or overwhelmed during the diagnostic and adjustment phases, which may lead to early discontinuation of therapy [24,25].

One particularly underexplored dimension involves the role of intimate relationships in shaping treatment outcomes. Concerns about the impact of CPAP on physical closeness, communication, and sexual intimacy are frequently cited by patients as barriers to regular use [24]. Importantly, OSAS is not an isolated experience; it often affects the sleep and well-being of the patient’s partner, who typically shares the sleeping environment. Disruptive symptoms such as loud snoring and nocturnal arousals can significantly disturb partners’ sleep, leading to daytime fatigue, irritability, and interpersonal strain [26,27]. These disturbances can create a feedback loop in which poor relational dynamics further reduce motivation to use CPAP consistently [26,27].

An emerging body of evidence suggests that partner involvement may play a critical role in encouraging and sustaining CPAP adherence. The patient–partner relationship—characterized by shared routines, mutual support, and emotional interdependence—can either serve as a facilitator or a hindrance to treatment adherence [28,29,30]. For instance, studies have found that patients who live with a partner tend to use CPAP more regularly than those who live alone, particularly during the initial stages of therapy. One study has reported that individuals cohabiting with a partner used CPAP for an average of 4.5 h per night in the first week, compared with 3.2 h among those living alone [31]. Another investigation found that CPAP adherence during the first two weeks was positively associated with the number of nights couples spent sleeping together [32].

While engaging spouses or bed partners has been suggested as a way to enhance CPAP adherence [33], the extent to which partner support influences treatment adherence remains insufficiently understood. There is a clear need to explore how partners contribute to or detract from the process of initiating and maintaining therapy, and to identify the emotional, relational, and contextual factors that shape these dynamics. Understanding these elements is essential for three reasons: (1) supporting individual patients; (2)addressing the needs of the couple as a unit, thereby optimizing long-term outcomes; (3) informing professionals on how to personalize treatment and focusing particular attention on patients who lack of adequate partner support.

Despite the growing recognition of the dyadic nature of sleep and illness management, no comprehensive synthesis of the literature has been conducted to date on partner involvement in CPAP adherence.

The primary goal of this systematic review is to address the existing gap in the literature by providing a comprehensive synthesis of empirical research on the role of partner involvement in both the initiation and long-term adherence to CPAP therapy among individuals with OSAS. Specifically, this review aims to outline the key characteristics of studies that have explored how relational factors influence CPAP adherence and to examine how the presence and support of a partner might affect patients’ consistency in using CPAP. Furthermore, the review seeks to investigate the impact of both relational facilitators and barriers on adherence to CPAP therapy. In addition, how relational factors and CPAP adherence are typically measured and assessed within the existing research is considered.

By pursuing these aims, this review ultimately hopes to underscore the importance of including relational aspects in the initial evaluation of CPAP therapy outcomes. Doing so may enable clinicians to deliver more personalized and effective interventions that take into account the dynamics of the patient’s support network, thereby improving both adherence to therapy and overall patient and family well-being.

## 2. Materials and Methods

This review was conducted following the Preferred Reporting Items for Systematic Reviews and Meta-Analyses (PRISMA) guidelines [34] (refer to Supplementary Materials Table S1) and was prospectively registered in the PROSPERO database for systematic reviews (registration number: CRD420251016574).

### 2.1. Search Strategy

In March 2025, relevant literature was identified using PubMed, Scopus, Medline, PsycINFO, and Web of Science. The search strategies utilized a combination of key terms and medical subject headings (MeSH) structured according to the PICO framework (Patient/Population, Intervention, Comparison/Control, Outcome) [35]. Specifically, studies were included if they focused on adults with OSAS (P) receiving CPAP therapy (I) who were either in a couple relationship or, when possible, compared with single individuals (C) and which assessed the influence of partner involvement or support on CPAP adherence or usage (O). Boolean operators and truncation were applied to systematically broaden the search and capture variations of search terms, using combinations such as: (“OSAS” OR “Obstructive Sleep Apnea Syndrome”) AND (“CPAP” OR “Continuous Positive Airway Pressure”) AND (“Partner involvement” OR “Partner participation” OR “Couple” OR “Partner support”) AND (“Adherence” OR “Compliance” OR “CPAP use”).

The search syntax was modified as appropriate for each database.

### 2.2. Inclusion and Exclusion Criteria

Eligibility criteria were established to include only original research articles that (1) were published in English, (2) provided full text access, (3) examined the effect of partner involvement or support on CPAP adherence or usage as the main outcome, and (4) focused on adult individuals diagnosed primarily with OSAS. Studies were excluded if they (1) were duplicate publications, preprints, grey literature, conference abstracts, or other systematic reviews and meta-analyses; (2) did not specify participant age, or had a sample with a mean age under 18 years; (3) lacked a clearly defined outcome or did not present enough detail to determine if the outcome directly related to the treatment; and (4) did not involve human participants (e.g., opinion articles, commentaries, or protocol papers).

No restrictions were set for publication date or study design, enabling the inclusion of randomized controlled trials, pilot studies, and qualitative research. While review articles and meta-analyses were not included in the analysis, their reference lists were examined for potentially eligible studies; however, no additional relevant articles were found.

### 2.3. Study Selection

Following the completion of the database search and the removal of duplicates, two reviewers (G.R. and C.C.) independently assessed study eligibility. The evaluation process involved an initial screening of titles and abstracts, followed by a full-text review according to the predetermined inclusion criteria. Any disagreements were resolved by a third author (F.C.). Additionally, the reference lists of all included studies and relevant systematic reviews were manually reviewed to identify any further studies for potential inclusion, though no additional articles were found. Consistent with the guidance provided by Smith et al. [36], the review team comprised two members (G.R. and G.P.) with methodological expertise in systematic review procedures, along with at least two subject matter experts (G.R., G.P., and G.C.), ensuring both methodological rigor and domain-specific insight throughout the review process.

The electronic database search yielded 121 records. After removing duplicates and screening titles and abstracts, 70 articles were excluded. The remaining 16 full-text articles were assessed for eligibility, excluding 7 studies [27,37,38,39,40,41]. In total, 9 studies met the inclusion criteria and were included in the final review [24,42,43,44,45,46,47,48,49].

**Figure 1:**
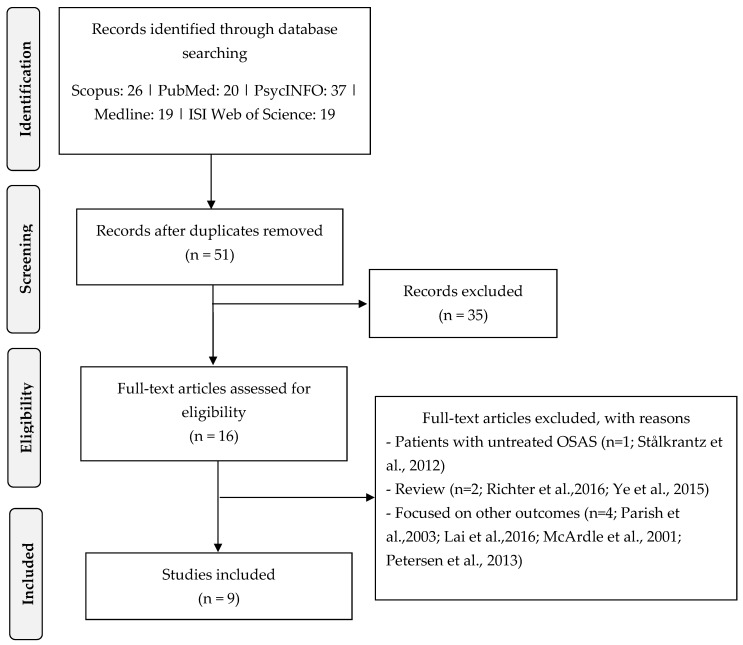
Flowchart of the study selection [27,37,38,39,40,41,50].

Following the PRISMA guidelines [34], the flowchart presented in Figure 1 provides step-by-step details of the study selection process.

### 2.4. Assessment of Methodological Quality

The mixed methods appraisal tool (MMAT-v2018) [51] was selected to assess the methodological quality of the included studies, due to its suitability for evaluating both quantitative and qualitative research designs. The MMAT requires reviewers to respond to each quality criterion using a categorical scale: “yes”, “no”, or “can’t tell”. For each study, an overall quality score out of five was assigned based on the total number of criteria rated as “yes” [52]. The assessment criteria addressed several aspects, including the suitability of the study’s methodological approach, the rigor of data analysis and collection techniques, the representativeness of the study sample, the reliability of outcome data, and the validity of the authors’ interpretation of results. The quality appraisal was conducted independently by C.C., F.C., and M.R. Any disagreements were discussed among the reviewers, and when consensus could not be reached, a third reviewer (G.R.) was consulted.

### 2.5. Data Extraction and Synthesis

Data extraction was performed independently by three authors (C.C., F.C., and M.R.), who collected information from each study, including (1) first author and year of publication, (2) country, (3) study design, (4) study aim, (5) follow-up points, (6) sample characteristics, (7) outcome measures, (8) drop-out rate, and (9) main findings. Any discrepancies encountered during the extraction process were resolved through discussion, with input from a fourth author (G.R.) as needed to achieve consensus. The extracted data were then synthesized to produce a narrative summary of the studies included in the review.

## 3. Results

### 3.1. Methodological Quality of the Included Studies: The Mixed Methods Appraisal Tool

Overall, the studies showed an acceptable level of methodological rigor. Five studies [42,45,47,48,53] obtained a score of 4 out of 5, indicating good methodological quality, although not without some limitations—primarily related to incomplete outcome data or unclear handling of missing information.

Two studies, both qualitative in study design [24,49], achieved the maximum score of 5/5, reflecting high methodological quality and comprehensive reporting across all criteria. These studies demonstrated strong adherence to qualitative research standards, including transparency in data collection and analysis, as well as appropriate consideration of researcher reflexivity.

Conversely, two RCTs [44,46] received the lowest scores (2/5), mainly due to unclear or unmet criteria regarding participant selection, measurement tools, and management of confounding variables. These lower scores suggest potential risks of bias and limitations in the interpretation of their findings. Results are reported in Table 1 below. Overall, although quantitative studies frequently encounter issues related to incomplete data and insufficient reporting of participant dropouts, qualitative studies [24,49] tend to exhibit stronger methodological rigor, offering valuable and reliable insights within MMAT-based research assessments.

### 3.2. Characteristics of the Included Studies

Details of the nine included articles are provided in Supplementary Materials Table S2. The selected articles were published between 2017 [43,44,48,49] and 2022 [24,46], and were conducted in the United States (n = 4) [43,44,46,49], France (n = 2) [45,49], Italy (n = 2) [24,48], and Canada (n = 1) [42]. In terms of study design, two studies adopted a qualitative approach [48,54], three were observational studies [45,47,49], one followed a cohort design [42], and two were randomized controlled trials (RCTs) [43,46].

### 3.3. Description of the Sample

The selected studies included 1,024 patients diagnosed with OSAS, comprising both genders, with a predominance of males (n = 738). Sample sizes varied considerably, ranging from a minimum of 20 participants [43] to a maximum of 290 [45,49] across studies. The overall mean age of participants was 56.68 years, with ages ranging from 37 [43] to 77 years [24]. Information on educational level and current patient employment was lacking among the included studies; in fact, only three studies described patients’ educational status [24,46,49], and only two described current patient employment [45,47].

The majority of the participants with OSAS in the included studies showed a body mass index (BMI) in the obesity class I (BMI: 30.0–34.9 kg/m^2^) [24,44,45,47] or in the obesity class II (BMI 35.0–39.9 kg/m^2^) [24,42,43,46]. Two studies did not report the BMI of the sample [48,49]. The average apnea–hypopnea index (AHI), which reflects the frequency of apneas and hypopneas occurring during sleep, was documented in eight of the nine included studies [42,43,44,45,46,47,48,49] and had an average value of 29.9. The Epworth sleepiness scale (ESS) [55], a self-report tool used to evaluate daytime sleepiness, had an average value of approximately 9.86 in eight of the nine studies reviewed [42,43,44,45,46,47,48,49].

Only five studies [42,45,47,48,49] examined the duration of the participants’ romantic relationships. The minimum relationship duration reported was six months [42]. In Tramonti et al. [48], the median relationship length was 34.7 years, while Ye et al. [49] reported a median of 16 years. Furthermore, three studies [45,47,49] also assessed the duration of cohabitation, with median values of 25 years [45,47] and 15.9 years [49].

### 3.4. The Impact of Relationship Status and Co-Sleeping on CPAP Adherence

Among the nine included studies, several relational factors were identified as influencing adherence to CPAP therapy. In two studies [43,45], the presence of a partner was found to promote greater CPAP adherence. Specifically, partnered individuals exhibited higher average nightly CPAP usage measured with a software device when compared with their unpartnered counterparts. For instance, in Baron et al. [43], applying the U.S. Centers for Medicare and Medicaid Services (CMS) adherence criteria (at least 4 h per night on 70% or more of nights during the first 90 days), 40% of individuals with a partner and all single participants were classified as non-adherent.

Gentina et al. [45] further demonstrated that bed-sharing positively influenced CPAP adherence in male patients.

Mendelson et al. [47] also explored spousal involvement by assessing how frequently partners engaged in challenges related to OSAS management. Their results show that younger couples reported higher levels of spousal involvement compared with mature and retired couples, although this difference was not statistically significant. Interestingly, older retired couples demonstrated the highest levels of CPAP adherence compared with both younger and mature couples.

### 3.5. Relational Quality Influencing CPAP Adherence

In the nine included studies, only five studies [42,43,44,45,48] explored the impact of relationship quality on CPAP adherence using validated tools such as the Couples’ Satisfaction Index (CSI-16) [42], Quality of Relationship Inventory (QRI), and Enhancing Recovery in Coronary Heart Disease Patients Social Support Index (ESSI) [43], Quality of Marriage Index (QMI) [45], and Dyadic Adjustment Scale (DAS) [48]. One study [42] reported a positive correlation between couple satisfaction and both the average nightly duration of CPAP usage and the frequency of nights exceeding four hours of use, as per adherence guidelines. However, this association did not persist at the 3-month follow-up, where no significant association was observed between CSI-16 scores and CPAP adherence. Notably, relationship satisfaction remained stable across time points (T0 and T3), with no significant changes detected.

The study by Baron et al. [43] also indicated that relationship status and relationship quality were significant predictors of CPAP adherence among women, while perceived relationship support did not show a significant impact on CPAP daily usage.

Gentina et al. [45] also found that higher marital quality (assessed via the QMI) was associated with greater CPAP adherence and that long-term relationships—lasting 30 years or more—acted as an independent factor promoting adherence. The same study [45] noted that spousal engagement during the initial CPAP adjustment phase significantly contributed to higher adherence levels. This finding is echoed in another study [44], which showed that increased spousal involvement was linked to higher adherence—particularly among men—in the CPAP group (as compared with a control group receiving minimal or ineffective airflow). Interestingly, this relationship was not maintained after a three-year follow-up, where no association between spousal support and self-reported adherence was found, even after stratifying by gender. However, it is important to note that, at three-year follow-up, a self-report measure of CPAP adherence was used, which could have influenced the objectivity of the results.

Tramonti et al. [48] showed that higher relationship satisfaction is associated with greater CPAP adherence among patients treated for OSAS, compared with those who are untreated. This increased adherence contributes to improvements in sleepiness, as measured by the ESS.

Furthermore, these findings are supported by two additional qualitative studies [24,49] that offered deeper insight into relational facilitators and barriers. Facilitators included active partner involvement during diagnosis and treatment, shared responsibility in CPAP use (joint effort), mutual sleep benefits, motivation driven by a desire not to disturb the partner, and emotional or practical partner support. Conversely, reported barriers encompassed anxiety about using CPAP, the disruptive nature of the equipment, interruptions in intimacy, and concerns related to appearance while wearing the mask.

Only one study [43] explored the effect of relationship conflict, measured via the QRI and ESSI, and reported a negative correlation between relationship conflict and CPAP adherence.

**Figure 2:**
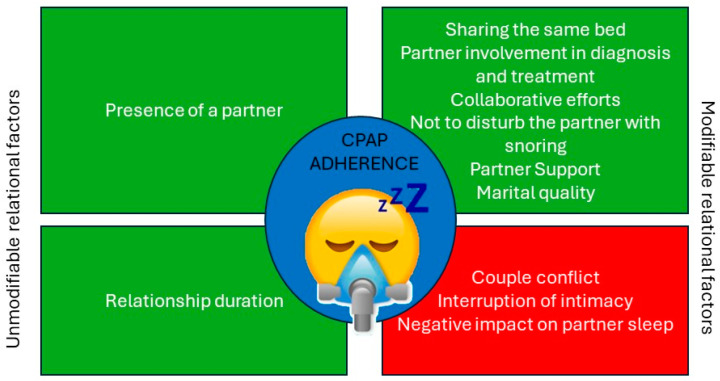
Barriers and facilitators of adherence to CPAP.

Figure 2 illustrates the relational barriers and facilitators of CPAP adherence identified in the included studies.

### 3.6. Effects of Relational Variables on Secondary Outcomes

The influence of relational dynamics on secondary outcomes emerged in several studies. In one study [43], greater relationship conflict was significantly associated with higher levels of depressive symptoms and increased cognitive and somatic pre-sleep arousal among patients with OSAS using CPAP. The same study [43] also revealed that partnered participants reported lower scores on the Insomnia Symptoms Index (ISI) compared with their unmarried counterparts, suggesting a potential protective effect of relational support on sleep quality. Baron et al. [43] analyzed qualitative data on spousal behaviors and found that partner encouragement positively affected CPAP adherence. They identified eight forms of partner supportive actions: asking about CPAP use, assisting with problem-solving, providing emotional support, offering encouragement, using humor, helping the patient recognize the benefits of therapy, reducing self-consciousness, and checking for snoring during sleep. Additionally, Khan et al. [46] employed the CPAP Tactics Survey to evaluate spousal participation from both the patient’s and caregiver’s perspectives. From the patient’s perspective, notable differences emerged between the intervention and control groups over time for item 7 (“Told me they were happy I was using CPAP”) and item 15 (“Discussed using CPAP”). In contrast, from the caregiver’s perspective, none of the 25 strategies evaluated demonstrated statistically significant differences at either the 3- or 6-month follow-up, or across the entire study period.

## 4. Discussion

The primary aim of this systematic review was to synthesize and critically examine existing evidence on the influence of relational factors—particularly partner involvement and relationship quality—on adherence to CPAP therapy in patients with OSAS. While individual factors such as disease severity, perceived benefit, and device comfort have been well documented in the literature as predictors of CPAP use, this review highlights an often-overlooked dimension: the social and relational context in which treatment occurs. This focus enhances our understanding of the patient profiles associated with better or worse CPAP adherence. As highlighted in the literature, personalized interventions are considered the gold standard for achieving cost-effective outcomes [56]. However, while a limited number of studies have explored tailored approaches based on patients’ socio-demographic characteristics [57,58], none have incorporated relationship status or the quality of relational dynamics as factors for personalizing care. This represents a significant gap in current clinical practice and research.

### 4.1. The Impact of Relationship Status and Co-Sleeping

The reviewed studies point to a clear association between being in a committed relationship and better adherence to CPAP therapy. One study [47] also demonstrated that couple age and employment should influence CPAP adherence because retired couples are more engaged in CPAP adherence than younger couples. This highlights that couples still at work could be more stressed about the situation and the adjustment to CPAP, and so clinicians should give more attention to this cluster of couples. Married or partnered individuals exhibited significantly higher levels of usage compared with their unpartnered counterparts [43,45]. These findings align with the broader literature on chronic disease management, which consistently shows that patients with supportive partners have improved health behaviors and outcomes, particularly in conditions requiring long-term adherence (e.g., diabetes, cardiac rehabilitation) [59,60,61].

Notably, the effect of co-sleeping emerged as a specific relational factor influencing adherence, particularly among male patients. Sharing a bed may reinforce a sense of accountability, provide emotional reassurance, or serve as a motivator to reduce sleep disturbances for the partner. This finding parallels earlier work on sleep concordance, where synchronized sleep routines and shared sleep spaces contribute to better treatment engagement [62].

### 4.2. Partner Support as a Facilitator—And Sometimes a Barrier

Beyond relationship status alone, the quality of the relationship plays a critical role. Higher levels of couple satisfaction, as assessed through tools like the CSI-16, the DAS, and the QMI, were associated with greater nightly use of CPAP and adherence to clinical guidelines [42,43,45]. These results suggest that emotional closeness and positive communication may buffer against the discomfort, stigma, or lifestyle changes imposed by CPAP therapy. Interestingly, some studies highlighted that spousal involvement was only beneficial when the underlying relationship quality was already high. For example, Gentina et al. [45] found that partner engagement in CPAP use predicted adherence only among couples with elevated QMI scores. This underscores the importance of considering not only the presence of partner involvement but also the quality and type of that involvement.

Studies in the review demonstrated that emotional and practical support from the partner—such as encouragement, collaborative problem-solving, and reducing embarrassment—significantly enhances CPAP adherence [43,45,49]. In qualitative analyses, couples engaging in joint advocacy (e.g., sharing responsibility for remembering or adjusting the device) were more likely to integrate CPAP into daily life [24,49] successfully. In relationships marked by high satisfaction, support tends to be perceived as caring and helpful, fostering better patient health outcomes. On the other hand, even well-intentioned involvement can be counterproductive in relationships strained by conflict or low intimacy. These findings emphasize the need to pay greater attention to relational dynamics when designing interventions aimed at improving CPAP adherence. These results are consistent with the wider literature on chronic illness management, which shows that patient outcomes tend to improve when partners provide supportive—rather than controlling or judgmental—interactions [63]. In contrast, relational conflict or ambivalence has been shown to undermine treatment adherence and emotional well-being.

For example, Baron et al. [43] found that relationship conflict was significantly correlated with poor adherence and increased psychological distress, including depressive symptoms and pre-sleep arousal. This is consistent with prior findings in health psychology, where overprotective or controlling behaviors from partners have been linked to reduced self-efficacy and autonomy in managing illness [64,65,66,67].

### 4.3. Limitations and Strengths of This Study

These findings should be interpreted with caution due to several limitations. First, the review included only original articles published in English, which may have led to the exclusion of relevant studies published in other languages. Moreover, gray literature was not considered; although this decision supports the inclusion of peer-reviewed, high-quality research, it may have limited the comprehensiveness of the review, as some relevant but unpublished or non-peer-reviewed studies were excluded. Despite these limitations, the review has several noteworthy strengths. Its focused examination of relational variables and their impact on CPAP adherence addresses a significant gap in the existing literature, emphasizing the importance of social and relational dynamics in the management of OSAS. Additionally, the inclusion of diverse study designs—ranging from qualitative and observational to cohort studies and randomized controlled trials—contributes to the overall methodological richness of the review. This variety enhances the robustness of the findings and allows for a more comprehensive understanding of the topic from multiple research perspectives.

### 4.4. Suggestion for Further Studies

The role of the partner in CPAP therapy represents a promising yet underexplored area of research that warrants further investigation, particularly through RCTs. Of the studies included in this review, only two utilized an RCT design, limiting the ability to draw strong causal inferences regarding the impact of partner involvement on CPAP adherence and treatment outcomes. Conducting rigorously designed RCTs would provide more conclusive evidence on the efficacy of partner-focused interventions and help establish best practices for incorporating partner support into CPAP therapy programs.

Moreover, the active inclusion of partners in both treatment protocols and research designs remains limited across the existing literature. Most of the reviewed studies failed to directly involve partners, thus constraining our understanding of the dyadic influences on adherence and outcomes. Integrating partners as active participants in CPAP-related studies would offer a richer understanding of how relational dynamics affect patient behavior, thereby enhancing both theoretical knowledge and practical strategies for intervention. A dyadic research approach not only adds depth to empirical findings but also provides actionable insights for optimizing CPAP adherence within the relational context.

A further limitation of the current evidence is the lack of adequate representation of female patients. With only one study [43] specifically focusing on women with OSAS, the influence of male partner support remains largely unexamined. This gap is particularly concerning, as relational dynamics and adherence behaviors may differ significantly by gender. Future studies should aim to explore the specific ways in which male partners influence treatment adherence in women, in order to identify gender-specific facilitators and barriers.

In addition, one study [45] examined partner support in the context of same-sex versus heterosexual couples, pointing to an important gap in inclusivity. Future research should prioritize diversity in participant recruitment to better understand how partner support functions across different relationship types and demographic groups. Addressing these gaps is essential for developing more equitable and generalizable CPAP adherence interventions.

Future research should consider other relational elements that could mediate or moderate the association between relationship quality and CPAP adherence, for example, exploring the role of patients’ and partners’ illness perception [68].

Notably, the studies included in this review were conducted exclusively in Western countries, where individualistic values and specific relational dynamics may influence both the provision and perception of partner support. In fact, perceptions of support within intimate relationships are deeply influenced by cultural norms and social contexts. What is considered helpful or supportive in one cultural setting may be perceived as intrusive or inappropriate in another. These cultural frameworks shape expectations around caregiving roles, emotional expression, and interdependence, all of which can significantly impact how support is given and received in the context of long-term treatments such as CPAP therapy. As such, further research is needed to explore these dynamics in non-Western contexts, where cultural values and social structures may lead to different patterns of support and adherence behavior.

Lastly, while this review included studies of varying methodological designs and strengths, several limitations in reporting and study quality were noted. Incomplete data, a lack of important socio-demographic information like educational level and current employment as well as clinical indicators, unclear sampling methods, and variability in outcome measures reduce the reliability of some findings. Furthermore, this high heterogeneity and the missing information do not contribute to the assessment and exploration of which patient and partner characteristics are associated with CPAP adherence in order to analyze elements of personalized care. Moving forward, researchers should strive for greater methodological rigor and transparency to improve replicability and enhance the overall quality of evidence in this field.

### 4.5. Clinical Implications and Recommendations

The results of this systematic review demonstrate the importance of relational factors—particularly partner involvement and relationship quality—in influencing adherence to CPAP therapy among individuals with OSAS. Building on these findings, we propose a series of recommendations for clinical practice, organized according to the intensity of intervention required, to improve CPAP adherence through relational and couple-based approaches.

First of all, it is essential to promote awareness-raising initiatives for medical and psychological staff working with OSAS patients, emphasizing the partner’s crucial role as a caregiver in OSAS management and CPAP adjustment. Clinical interventions and follow-up care should be tailored to individual circumstances, with particular attention given to patients who live alone or whose partner involvement may be limited or counterproductive. Notably, when support is perceived as pressure or intrusion, it can generate resistance and reduced adherence. Furthermore, clinicians should be trained to identify potentially negative relational dynamics during their visits by asking questions about relational quality to patients and, when appropriate, involving both the patient and the partners to see how they interact in practice. This approach facilitates a more holistic understanding of the interpersonal context influencing treatment adherence

Regarding the couple-based approach, at the most basic level, low-intensity interventions should focus on educating both patients and their partners about the importance of CPAP in managing OSAS and preventing long-term health complications. Providing accessible information about the benefits of therapy and the critical role of partner support can help lay the foundation for shared engagement. Encouraging open communication between partners is also essential—discussing concerns, expectations, and practical strategies to support daily use of the CPAP device can make a meaningful difference in adherence.

Moving toward more structured interventions, healthcare professionals—particularly psychologists and sleep specialists—can organize joint training sessions for couples. These sessions can cover the technical aspects of CPAP use, such as setup, maintenance, and troubleshooting, while also promoting active partner involvement. Creating opportunities for couples to learn together reinforces the idea that CPAP adherence is a shared effort. In addition, support groups specifically tailored for couples managing CPAP therapy can offer a safe space for sharing experiences, discussing challenges, and providing mutual support. Incorporating stress management techniques—such as guided relaxation, breathing exercises, or mindfulness practices—can also help couples cope with emotional stressors related to the use of CPAP. Encouraging partners to attend education sessions or participate in treatment discussions may strengthen adherence.

In cases where relationship difficulties or emotional barriers affect treatment, higher-intensity interventions may be appropriate. Couples therapy or relationship counseling can be highly effective in addressing interpersonal conflicts, improving communication, and strengthening the emotional bond between partners. These sessions can focus on enhancing mutual understanding, resolving tensions, and jointly navigating the challenges of CPAP therapy. Additionally, behavioral strategies—such as goal setting, positive reinforcement, and collaborative problem-solving—can be employed to help couples develop practical routines that support long-term adherence.

It is also important to emphasize the need for individualized interventions, tailored to each couple’s unique context. Factors such as relationship duration, lifestyle, communication patterns, and mutual expectations should inform the design of any support plan. Continuous monitoring and follow-up are equally essential, allowing clinicians to track adherence, address emerging barriers, and reinforce successful strategies over time.

Ultimately, by involving partners in the therapeutic process and acknowledging the relational context of CPAP use, healthcare professionals can adopt a more holistic and effective approach to managing OSAS. These interventions not only support adherence but may also improve relationship quality, emotional well-being, and overall quality of life for both patients and their partners.

## 5. Conclusions

These findings highlight the multifaceted impact of relational factors on CPAP adherence in patients with OSAS. Elements such as the presence of a partner, especially a supportive one with whom there is a satisfying and mutually supporting relationship, significantly contribute to consistent CPAP use and, consequently, to better treatment outcomes. At the same time, it is essential to recognize and address potential relational barriers—such as anxiety, communication difficulties, or interpersonal conflict—that can undermine adherence and negatively affect patient well-being. Continued research into these relational dynamics, along with the development of targeted interventions to enhance partner involvement, holds great promise for improving CPAP adherence and advancing patient-centered care in the treatment of OSAS.

## Figures and Tables

**Table 1:** MMAT quality assessment.

Author (Year)	Study Design	Criterion 1	Criterion 2	Criterion 3	Criterion 4	Criterion 5	Total ✅	Methodological Quality
Adams et al., 2020 [42]	Quantitative descriptive	✅	✅	✅	❌	✅	4/5	Acceptable/High
Baron et al., 2017 [43]	Quantitative descriptive	✅	✅	✅	❌	✅	4/5	Acceptable/High
Batool-Anwar et al., 2017 [44]	Quantitative RCT	✅	❌	❌	✅	❌	2/5	Low
Gentina et al., 2018 [45]	Quantitative descriptive	✅	✅	✅	❌	✅	4/5	Acceptable/High
Khan et al., 2022 [46]	Quantitative RCT	❓	❓	✅	❓	✅	2/5	Low
Mendelson et al., 2020 [47]	Quantitative descriptive	✅	✅	✅	❓	✅	4/5	Acceptable/High
Rapelli et al., 2022 [24]	Qualitative	✅	✅	✅	✅	✅	5/5	High
Tramonti et al., 2017 [48]	Quantitative descriptive	✅	✅	✅	❓	✅	4/5	Acceptable/High
Ye et al., 2017 [49]	Qualitative	✅	✅	✅	✅	✅	5/5	High

**Note**: Criterion 1. Is the sampling strategy relevant to address the research question? Criterion 2. Is the sample representative of the target population? Criterion 3. Are the measurements appropriate? Criterion 4. Is the risk of nonresponse bias low? Criterion 5. Is the statistical analysis appropriate to answer the research question? ✅ Yes; ❌ No; ❓ Can’t tell.

## Data Availability

No new data were created or analyzed in this study.

## Supplementary Materials

The following supporting information can be downloaded at https://www.mdpi.com/article/10.3390/jpm15050192/s1, Table S1: Prisma 2020 Checklist, Table S2: Characteristics of included studies.
