# Supplementary material for: Three in a Bed: Can Partner Support Improve CPAP Adherence? A Systematic Review and Intervention Recommendations
Source: J Pers Med. 2025 May 8;15(5):192. doi: 10.3390/jpm15050192 (PMC12113088; doi:10.3390/jpm15050192)
Supplement: Supplementary file 1 [file jpm-15-00192-s001.zip › jpm-3596214-Table S2.pdf]

Table S2. Characteristics of included studies

Author, year	Country	Study design	Study aim *	Follow-up points**	Sample (n) Patients: Partners	Age (years: Mean; SD; range) Patients: Partners	Patient Educational level: n(%)	Patient employment: n(%)	Patient gender: *n(%)*	BMI (kg/m²): *mean(SD), range*	ESS AHI (SD) (events/h)	Couple's relationship duration (years)
Adams et al., 2020 [42]	Canada	QT, cohort design	Examine the role of **individual attachment** and **couple satisfaction** on **compliance with CPAP treatment**. It is also investigated the **benefit of CPAP treatment** on sleep and anxiety and depression.	**T0-T3**	N = 31	50.6 (± 9.4)	NR	NR	Male patients: 8 (25.8%)	35.43 (7.30)	**ESS** = 9.71 (5.48) **AHI** = 34 (18.30 ± 60.60)	NR (6 months relationship)
Baron et al., 2017 [43]	Chicago, USA	QT, pre-post prospective study	Evaluate the role of **relationship quality** in women's **continuous positive airway pressure** (CPAP) **adherence.**	**T0 - T3**	N=20	50 (± 10)	NR	NR	Female patients: 20 (100%)	37.8 ± 10.8	**ESS** = 11.7 ± 5.0; **AHI** = 20.5 ±16.3 events/h	NR
Batool-Anwar et al., 2017 [44]	USA	QT, Randomized Control Trial	To determine whether **spouse involvement** (measured by DAS) affects **CPAP adherence** and how this association differs by gender using data from a large randomized trial of CPAP versus sham CPAP to treat OSAS.	**T0-T2-T6-T36**	N =194 (**Compliant** N = 82; **Non-compliant** N = 112; Sham-CPAP[§] N = 84; CPAP N = 110)	56.15 (9.77) Compliant-Sham 59.6 (9.9) Compliant CPAP 59 (8.7) Non-compliant Sham 53 (10.5) Non-compliant CPAP 53 (10)	NR	NR	Male patients: 143 (73%) Compliant Sham 19 (68%) Compliant CPAP 45 (83%) Non-compliant Sham 39 (69%) Non-compliant 40 (71%)	31 (6.6) Compliant Sham 31 (6.9) Compliant CPAP 31.3 (5.2) Non-compliant Sham 32 (7.7) Non-compliant CPAP 31.4 (6.6)	**ESS**: NR **AHI**: ≥ 10 (10.0 ± 15.0 events per hour) (Inclusion criteria)	NR

Gentina et al., 2018 [45]	France	QT, multicenter prospective observational study	Assess the impact of **relationship quality, partner's engagement** and **couple's characteristics** (duration of the couple, number of children, sharing the same bed) on **CPAP adherence**.	**T0-T4**	N= 290	53 (46 ; 62)	NR	Active 188 (65.5) Working at home 6 (2.1) Unemployed 14 (4.9) Retired 79 (27.5)	Male patients: 224 (77.2%)	32 (28.6 ; 35.9)	**AHI**: 43 (33 ; 58);<br><br>**ESS**: 11 (6 ; 15)	25 years* (13; 35.5)<br><br>*median; duration of living together
Khan et al., 2022 [46]	USA	QT: Randomized controlled parallel group clinical trial with intervention and control arms **§**<br><br>PILOT STUDY	Determine the impact **of a multidimensional treatment framework** (based on shared decision-making, patient activation, and caregiver engagement) on improving **long-term CPAP adherence** in patients newly diagnosed with OSAS.	T0-T3-T6	N= 60 (Intervention Group n = 28 patients + 28 caregiver; Control group n = 32 patients + 32 caregiver)	53.5 (± 12.97)<br><br>Intervention group: 58 (± 11.75) Control group: 50 (± 12.92)	High school graduate or below: 11 (19); Some college or above: 48 (81)	NR	Male: 23 (38%)<br><br>Intervention group: 12 (43%) Control group: 11 (34%)	37.6 ± 9.84<br><br>Intervention group: 35.5 ± 7.2 Control group: 39.4 ± 11.5	**ESS (tot baseline: 9,65)**: Intervention group baseline: 9.1 ± 0.9 Intervention group 3-months: 6.6 ± 0.95 Intervention group 6-months: 6.0 ± 0.7 Control group baseline: 10.2 ± 0.9 Control group 3-months: 6.5 ± 0.8 Control group 6-months: 4.8 ± 0.7 (p-value 0.11)<br><br>**AHI**: 26.0 ± 23.0 (p-value = .83) Intervention group: 26.8 ± 25.0 Control group: 25.5 ± 21.6	NR

Mendelson et al., 2020 [47]	France	QT, multicenter prospective study	Identifying specific **clusters of OSAS couples** and their association with **CPAP adherence** 120 days after CPAP initiation.	T0-T4	N = 290<br>Class 1: Older Retired Couples n = 76 (26%)<br>Class 2: Young Working Couples n = 128 (44%)<br>Class 3: Mature Active Couples n = 86 (30%)	56<br>Class 1: 67 (63–71)<br>Class 2: 45 (40–49)<br>Class 3: 57 (54–59)<br>(p < 0.01)	NR	Class 1: both partners working 0(0); no one working: 73(97.3); one partner is working: 2(2.7).<br>Class 2: both partners working: 96(75); no one working: 0(0); one partner is working: 32(25).<br>Class 3: both partners working 43(53.1); no one working: 1(1.2); one partner is working: 37(45.7).	223 (77%)<br><br>Class 1: 60 (80%)<br>Class 2: 102 (79.7%)<br>Class 3: 62 (72.1%)	32 kg/m2 (28.6–35.9)<br>Class 1: 32.1 (27.6–35.7)<br>Class 2: 32.1 (28.9–35.8)<br>Class 3: 32.1 (27.7–37.4)	**ESS**: 10<br>Class 1: 7 (5-12)<br>Class 2: 12 (8–16)<br>Class 3: 11 (7-15)<br>(p < 0.01)<br><br>**AHI**: 43 (33–58)<br>Class 1: 42 (34.7–52)<br>Class 2: 43.2 (33–60.1)<br>Class 3: 44.2 (32–57)<br>(p-value 0.89)	25 years* (13–35.5)<br><br>*median

Rapelli et al., 2022 [24]	Italy	QL, focus group	To investigate the lived experience of patients with OSASS and comorbid obesity following after continuous positive airway pressure (CPAP) therapy	T0	32 patients	59.61; 11.18; 37–77 years	High School: 18(56.25); Bachelor's degree: 10(31.25); Master's degree: 4(12.5)	NR	16 (63%)	Obesity Class I (BMI 30.0–34.9 kg/m2): 22 Obesity Class II (BMI 35.0–39.9 kg/m2): 10	NR	NR
Tramonti et al., 2017 [48]	Italy	QT, observational study	To evaluate **dyadic adjustment** in a sample of treated or untreated patients with OSAS.	T0	N= 87 Treated with CPAP: 28 Untreated: 59	60.8<br><br>Treated: 63.4 (± 7.2) Untreated 58.2 (± 10.9)	NR	NR	71 males (81.6%)	NR	**ESS (tot: 7,15)**: Treated: 5.3 ± 4.0 Untreated: 9.0 ± 4.4<br><br>**AHI (tot:18,7)**: Treated: 6.3 ± 3.3 Untreated: 31.1 ± 22.2	**Relationship duration**: Treated: 37.2 ± 10.9 Untreated: 32.3 ± 15.3
Ye et al., 2017 [49]	USA	QL, exploratory qualitative descriptive design	To obtain a comprehensive qualitative description of **couples' experiences with CPAP treatment**, with a particular interest in determining facilitators and barriers to incorporating CPAP use into daily life.	T0	N = 40 (20 couples)<br><br>Patients = 20 Partners = 20	50 Patients = 49.6 (± 9.6) Partners = 50.1 (± 10.1)	College education: 17(85)	NR	14 patients male gender (70%)<br><br>Same sex = 4 (20%) Heterosexual =16(80%)	NR	**AHI**: 24.1 ± 18.5 **ESS**: NR	**Relationship duration**: 16 (2 - 49)<br><br>**Living together**: 15.9 - 12.8

(continue)

Author, year	Clinical outcomes: measure	Primary outcomes: measure	Secondary outcomes: measure	Drop-out N (%)	Results (clinical outcomes)	Results (Primary outcomes)	Results (Secondary outcomes)
Adams et al., 2020 [42]	**Sleep:** AHI, ESS, PSQI	**Attachment**: ECR; **Relationship quality**: CSI-16; **CPAP adherence**: software measure of compliance	**Depressive Symptom severity**: PHQ-9; **Anxiety Symptom severity**: GAD-7	N = 71 (69.7%)	**Sleep**: Compliance with CPAP treatment will improve sleep measures after 3 months: AHI (Z = - 4.86, p < .001,d=−1.19), PSQI (Z = - 4.57, p < .001), ESS (Z = - 9.94, p < .001).	**Attachment**: individuals who made a mutual decision with their partner had higher attachment anxiety [3.74 (1.20)] than those who self-prompted [2.87 (0.74), p=0.04]. There were no significant differences between individuals who self-prompted or were prompted by their partner (Mean difference = −0.37, p = .34) or partner-prompted individuals and those who made a mutual decision to seek treatment (Mean difference = − 050, p = .29). There were no significant differences between the groups for attachment avoidance (F (2,80) = 0.83, p = .44, f = 0.11). There were no statistically significant changes in attachment avoidance (p = .70), attachment anxiety (p = .37). There were no significant predictors of change in attachment.<br><br>**Relationship quality:** couple satisfaction was positively associated with the average number of hours the machine was used each night (F (1,29) = 5.94, p = .02). It was also a significant predictor of the percentage of nights the machine was used for ≥ 4 h (F (1,29) = 8.76, p < .01). There were no significant differences between the groups with respect to relationship satisfaction (p = .56). There were no statistically significant changes in CSI-16 (p = .98).	**Depressive Symptom severity**; **Anxiety Symptom severity**: There were statistically significant changes between T0 and T3 in: PHQ-9 (Z=-4.04,p<.001) and GAD-7 (Z = - 4.86, p<.001). Change in GAD-7 was statistically significant with the percentage of days the machine was used ≥4 has the sole predicting variable (F(1,29) = 6.18, p = .02). There were no significant predictors of change in depression, sleep quality, or daytime sleepiness over 3 months.

						**CPAP adherence: c**ompliance with CPAP treatment was significantly influenced by relationship satisfaction but not by attachment. Compliance with CPAP treatment was not significantly associated with CSI-16 scores at 3-month **follow-up** (F(1,29) =p = .26).	
Baron et al., 2017 [43]	**Sleep**: ESS; **Insomnia Symptoms**: ISI; **Apnea severity**: apnea severity index (events/hour)	**Relationship quality**: QRI; ESSI<br><br>**CPAP adherence**: CPAP devices	**Depressive Symptoms**: CES-D; **Pre-sleep Arousal**: Pre-sleep arousal scale; **Sleep Apnea Self-Efficacy**: Self-Efficacy Measure for Sleep Apnea; **Perceived importance of OSAS treatment:** one item	N=4	Comparing married and unmarried participants at T3:<br>ESS: 11.7 (4.7) vs. 11.7 (5.9)<br>ISI: 15.5 (7.9) vs. 20.4 (6.6)<br>Apnea severity: 16.2 (12.4) vs. 28.4 (20.5)	**CPAP Adherence**: at T3 the average adherence was 3.7 h (SD = 2.8; range = 0.1 h - 7.8 h). Of the 16 participants with adherence data, 6 (38%) were considered adherent (CPAP ≥4 h on ≥ 70% of nights).<br><br>There was a higher average nightly CPAP use among married/partnered participants compared to unmarried/unpartnered participants at T3: 4.6 h versus 2.1 h, p < 0.08. Unmarried/unpartnered participants reported greater CPAP self-efficacy than married participants at T3: 34.6 (4.6) vs 27.4 (6.7), p < .05). Using the Medicare adherence criteria of ≥4 h of use on 70% of nights, 40% of married or partnered participants and 100% of the unpartnered patients were non-adherent (p < .05)<br><br>**Relationship status and relationship quality strongly predict women's CPAP adherence**:<br>Among married or partnered patients, relationship conflict was negatively associated with adherence (r=-0.60, p<0.05) at T3. Greater perceived social support was positively associated with CPAP adherence among all participants (r = 0.65, p<0.05) at T3. Relationship support was not associated	**Cognitive and somatic pre-sleep arousal:** were associated with poorer CPAP adherence (r = -0.53, p < 0.05, r = -0.59, p < 0.05) and higher relationship conflict (r = 0.66, p < 0.05, r = 0.53, p < 0.05) at T3.<br><br>**Depressive symptoms** were correlated with relationship conflict (r= 0.58, p < 0.05), social support (r = -0.48,p < 0.05), cognitive (r = 0.60, p < 0.05) and somatic pre-sleep arousal (r = 0.63, p < 0.05) at T3.<br><br>Depressive symptoms, insomnia symptoms, CPAP self-efficacy were not associated with CPAP adherence.<br><br>6 participants reported support and encouragement from their spouse/partner to use CPAP. Helpful types of involvement included: asking about CPAP, problem-solving, support, providing encouragement, using humor, helping the participant "realize the benefits" of CPAP, encouraging use to "better my

						with CPAP adherence.	health", helping the participant to feel less self-conscious about it, and checking to see if she is snoring at night.
Batool-Anwar et al., 2017 [44]	**OSAS**: Polysomnography (EEG; EOG; EMG)	**Spousal Involvement**: DAS;<br><br>**Dyadic adjustment:** DAS;<br><br>**CPAP adherence**: nightly use of CPAP using software (at the 6-month follow-up visit).<br><br>**Long-term CPAP adherence**: self-reported adherence at the time of the DAS administration	NR	N= 112* (Sham N=56; CPAP N=56)<br><br>*Non-compliant	Over half of the participants had severe OSAS (62%)	**Spousal Involvement:** At T6, CPAP adherence was associated with advanced age (r = 0.015, p < 0.01) and increased spousal involvement (r = 0.07, p < 0.01). The association between CPAP adherence and spousal involvement was reported only for the CPAP group (r = 0.11; p=0.01) and in particular for men (r= 0.088, p=0.03).<br>3 years after APPLES, 82 participants were still adherent by self-report. At this time point, spousal involvement was not associated with CPAP adherence even after gender stratification (p < 0.13).<br><br>**Dyadic Adjustment**: Adjustment to marriage as reflected by items on the DAS questionnaire, however, was not associated with CPAP adherence.	NR
Gentina et al., 2018 [45]	**OSAS**: PSG or HST	**Relationship Quality**: QMI<br><br>**CPAP Adherence**: CPAP device's software	**Patient-reported disease-specific health-related quality of life**: ESS, QSQ<br><br>**Spousal involvement**: questionnaire evaluating partner's engagement with 3 main domains:	NR	**ESS**: at day 120, median CPAP adherence was 359 minutes [307; 425] per night and the ESS score had improved to a median value of 6 [3; 10]	**Relationship quality**:<br>Patients living alone exhibited poor CPAP adherence compared to those who were married or living as a couple. Sharing the same bed also has a positive effect on male OSAS patients' CPAP adherence. The quality of marriage index (QMI) revealed that the relationship between a partner's involvement and the patient's CPAP adherence was significant only for patients having a high QMI ($\gamma 4a = 0.32$; $p < 0.001$) but not	The **QSQ** improved between T1 and T4:<br><br>QSQ emotions 2.6 [2.0 ; 3.2] 1.8 [1.4 ; 2.4] <0.01<br>QSQ hypersomnolence 2.7 [1.8 ; 3.5] 1.5 [1.1 ; 2.2] <0.01<br>QSQ diurnal symptoms 3.0 [2.2 ; 3.6] 1.7 [1.2 ; 2.2] <0.01<br>QSQ nocturnal symptoms 2.7 [2.3 ; 3.4] 1.9 [1.4 ; 2.4] <0.01<br>QSQ social interaction 2.0 [1.3 ;

			pressure to use CPAP, emotional support, and collaboration in solving issues linked with CPAP usage.			for those with low QMI (γ4b = 0.02; p = 0.82).<br><br>**CPAP adherence** was significantly linked with disease-specific health-related quality of life improvement (γ2 = - 0.21, p < 0.05); the partner's engagement directly impacted improvement in disease-specific health-related quality of life in patients with OSAS (γ3 = - 0.15, p < 0.05). Encouragement of CPAP usage (p = 0.02) and duration of the couple's relationship >30 years (p = 0.01) remained independent factors for CPAP compliance at T4. The relationship between the partner's involvement and the patient's CPAP adherence was significant only for patients having a high QMI (γ4a = 0.32, p < 0.001) but not for those with low QMI (p = 0.82). In this specific sub-group (66 WOMEN), poor CPAP adherence at 120 days was mostly associated with active professional activity. Retired or inactive women exhibited better CPAP compliance at 120 days.	2.7] 1.3 [1.0 ; 1.7] <0.01<br><br>**Spousal involvement:** encouragement of CPAP usage (p=0.02) and duration of the couple's relationship >30 years (p=0.01) remained independent factors for CPAP compliance at T4. The relationship between the partner's involvement and the patient's CPAP adherence was significant only for patients having a high QMI (γ4a = 0.32, p < 0.001) but not for those with low QMI (p = 0.82).
Khan et al., 2022 [46]	NR	**CPAP adherence and mean daily use:** PAP with remote monitoring capabilities	**Sleep:** ESS<br><br>**Spousal involvement:** CPAP tactics survey<br><br>**Shared decision-making and the confidence in the decision made**: COMRADE survey	0	**ESS:** scores improved in both groups: Intervention group T0 (9.1 ± 0.9); T3 (6.6 ± 0.95); T6 (6.0 ± 0.7). Control group T0 (10.2 ± 0.9); T3 (6.5 ± 0.8); T6 (4.8 ± 0.7). (p = .11)	**CPAP Adherence and mean daily use:** In an age-adjusted model, the mean daily use of PAP increased significantly over the 3 time periods: Intervention group: from 4.2 (3.3–5.2) at T0 to 4.1 (3.1–5.2) at T3 and 4.6 (3.6–5.7) at T6, p = .03). Intervention group participants gained a mean of 1.23 hours in PAP mean daily use between 3 and 6 months vs those in the control group (P = .008).<br><br>In an age-adjusted model, the PAP adherence increased significantly over	**Spousal Involvement:** Patient perspective: only item 7 ("told me he/she was happy I was using CPAP", p= .01) and item 15 ("discussed using CPAP", p= .0008) had a significant change between the intervention and the control groups across time. For item 24 ("Gave me space, showed patience in order to get me to use CPAP"), the percentage of responding yes at 1 month was higher in the

						the 3 time periods:<br>Intervention group: from 46 (28-65) at T0 to 50 (32-68) at T3 and 54 (35-72) at T6, p=.46).<br><br>The changes between baseline and 3 months in the 2 groups were not statistically significant (P = .73). There was no difference in PAP adherence between the 2 groups (P = .63).<br>The adjusted changes between baseline and 3 months in the 2 groups were not statistically significant (P = .73).	intervention group than in the control group (P = .04).<br>Caregiver perspective: No significant differences in the 25 tactics were found either cross-sectionally at the 6-month follow-up or longitudinally across the 3-time points.**Shared decision-making and the confidence in the decision made**:<br>Item 7, item 8, item 10 and item 11 were found to be associated with the intervention (item 7 and item 8: odds ratio = 3.65, P = .02; item 10: odds ratio =3.25, P = .04).
Mendelson et al., 2020 [47]	**OSAS**: PSG or HST	**CPAP adherence**: CPAP device's software at day 120 **Spousal involvement (in CPAP use)**: Dyadic Coping scale (23 items) **Relationship quality:** DAS + QMI; SLEEP QUALITY: additional items of QMI	**Patient-reported disease-specific health-related quality of life**: ESS; QSQ and social and demographic variables	NR	**OSAS severity**: Patients in the older retired couples cluster presented the highest CPAP adherence (p < 0.01) independently of OSAS severity.<br><br>**ESS**: There was a significant difference in the decrease of the ESS for the cluster of young working and mature active couples compared to the older retired couples cluster. Compared to the older retired couples, the young working couples cluster presented a 2.84-point decrease of the ESS score (p < 0.01) and the mature	**QMI**: There was no significant change over time in the QMI in any couple clusters. At day 120 only the difference between the clusters of young working and older retired couples remained significant (retired couples cluster presented a lower QMI compared to the young couples cluster; median QMI: 18 (IQR: 16–21) vs. 20 (IQR: 18–22) vs. 20 (IQR: 17–22) in the older retired, young working and mature active couple clusters, respectively, p < 0.01).<br><br>**DAS**: There was a significant change in the DAS score between baseline and T4 between the clusters of older retired couples and young active couples. The median increase in DAS in the cluster of young active was 3 points (IQR: −1.5–7) while the cluster of older retired couples presented a median decrease of −2 points (IQR: −6–6).<br><br>**Spousal Involvement**: There were no	**QSQ**: The overall decrease in the total QSQ score was significantly greater for OSAS patients in the cluster of young working couples compared to the clusters of older retired and mature active couples [71 (IQR: 55–83) vs. 60 (IQR: 45–70), p < 0.01].

					active couples cluster presented a 1.64-point decrease of the ESS score (p = 0.04).	significant differences between the 3 couple clusters in term of spousal involvement in the CPAP therapy. There is a slight trend to a higher involvement for spouse's in the young couples cluster compared to the clusters of mature and retired couples, but this difference was not significant.<br><br>**Spouse's sleep quality**: The spouse's sleep quality was lower in the older retired couples cluster (moderate level: 3 (IQR: 2–3)) compared to the young/mature active couples cluster (low level: 2 (IQR: 2–2)).<br><br>**CPAP adherence**: the highest CPAP compliance in cluster older retired couples (6.6 h (IQR: 5.7–7.5) vs. 5.9 h (IQR: 4.9–6.6) vs. 5.9 h (IQR:159 4.9–7.3) for the retired, young and mature couple clusters, respectively, $p < 0.05$;). The use of an orofacial mask and couple clusters were associated with CPAP adherence. The use of an orofacial mask was associated with a significant decrease in 120-day CPAP adherence: 40 min (IQR: −68 to −12; p = 0.006).	
Rapelli et al., 2022 [24]	NP	NP	NP	NP	NP	Since many adults share sleep with partners, OSAS symptoms lead to daytime exhaustion in both partners and relationship issues. CPAP therapy improves sleep quality for both partners, restoring intimacy, but the device can also hinder intimacy due to its appearance and noise, impacting adherence to therapy.	NP
Tramonti et al., 2017 [48]	**Sleep**: ESS **Apnoea-hypopnoea**: AHI	**Dyadic Adjustment**: DAS	NR	NR	Treated patients had lower AHI and ESS scores (p=.000;	DAS total scores of the whole sample being close to local norms, untreated patients showed lower scores in the items: affective expression and spousal	NR

	**CPAP use:** data derived from the device logs				confirming the benefit of CPAP).	support (p = 0.046).<br>Age and relationship duration are positively correlated with total DAS scores in the CPAP-treated group ($p \leq 0.01$):<br>The ESS scores showed a negative correlation with many DAS scores (Dyadic satisfaction; Dyadic cohesion; Dyadic consensus; Affective expression; Dyadic adaptation, p<.01), whereas no significant correlation emerged for AHI.<br><br>Untreated patients showed lower scores on Affective Expression, Dyadic Consensus, and the DAS total scores when compared to the ones treated (treated dyadic consensus 55.1±10.4 vs. untreated 51.1±9.3, p = .012; treated affective expression 9.5±3.1 vs untreated 8.7±2.6, p = .044; treated dyadic adaptation 117.9±21.3 vs untreated 111.9±18.4, p.=046).<br><br>Age and relationship duration were positively correlated with the DAS total scores and the Dyadic Consensus subscale (p <.01). The ESS scores were negatively correlated with Dyadic Consensus, Affective Expression and Dyadic Satisfaction, as well as with the DAS total score.	
Ye et al., 2017 [49]	NP	**Couples' experiences of managing CPAP treatment together**: semi-structured interview	NP	NP	NP	**Facilitators of CPAP use**: partner aiding diagnosis and treatment, couples working together using CPAP (joint advocacy), benefits of CPAP for both partners (nice sleep), patient motivated to use CPAP for the benefit of the partner (not disturb), various types of support provided by the partner to encourage CPAP use.<br><br>**Barriers to CPAP use**: anxiety related	NP

			to CPAP use, bothersome equipment causing disruptions in sleep and bedtime routine, interruptions to intimacy, and concern about image change while wearing CPAP.	

Note: *Patient Reported Outcomes in bolt. **Psychological data measurements in bolt. NR: Not Reported; NP: Not Pertinent

§ The intervention group attended 4 structured sessions: interactive education, peer coaching, hands-on experience, and a semistructured motivational interview. The intervention group The control group was educated on physical activity and lifestyle only.

CES-D: Center for Epidemiologic Studies Depression Scale; COMRADE: Risk Communication and Treatment Decision-Making Effectiveness survey; CSI-16: 16-item Couples' Satisfaction Index; DAS: Dyadic Adjustment Scale; ECR: Experiences in Close Relationships scale; EEG: Electroencephalogram; EOG: Electro-oculogram; ESS: Epworth Sleepiness Scale; ESSI: Enhancing Recovery in Coronary Heart Disease Patients Social Support Index; GAD-7: Generalized Anxiety Disorder-7; HST: Home sleep test; ISI: Insomnia Severity Index; LTFU: Loss to follow-up on surveys; OSAS: Obstructive Sleep Apnea Syndrome; PHQ-9: Patient Health Questionnaire-9; PSG: Polysomnography; PSQI: Pittsburgh Sleep Quality Index; QMI: Quality of Marriage Index; QRI: Quality of Relationship Inventory; QSQ: Quebec Sleep Questionnaire; SHAM-CPAP: it is a control condition used in research studies on sleep disorders, particularly obstructive sleep apnea (OSA). It involves the use of a CPAP (Continuous Positive Airway Pressure) device that appears to function like a regular CPAP machine but does not deliver therapeutic pressure. Instead, it provides minimal or ineffective airflow. Sham-CPAP is employed to serve as a placebo control in clinical trials, allowing researchers to assess the true efficacy of CPAP therapy by comparing outcomes between the active treatment group (receiving genuine CPAP therapy) and the control group (receiving sham CPAP).
